# Clean Label “Rocha” Pear (*Pyrus communis L*.) Snack Containing Juice By-Products and *Euglena gracilis* Microalgae

**DOI:** 10.3389/fnut.2022.825999

**Published:** 2022-02-16

**Authors:** Xavier Lomba-Viana, Anabela Raymundo, Catarina Prista, Maria João Alegria, Isabel Sousa

**Affiliations:** ^1^LEAF - Linking Landscape, Environment, Agriculture and Food Research Centre, Departamento de Ciências e Engenharia de Biossistemas (DCEB), Instituto Superior de Agronomia da Universidade de Lisboa, Lisboa, Portugal; ^2^SUMOL+COMPAL Marcas S.A., Carnaxide, Portugal

**Keywords:** “Rocha” pear, clean label, food product development, *Euglena gracilis*, thick snack, pear pomace, by-products valorization

## Abstract

“Rocha do Oeste” pear is a Portuguese Protected Designation of Origin variety and one of the country's most relevant fruits for its nutritional value, production area, and exportation amounts. The recent integration of a pilot-scale juice production line brought to SUMOL+COMPAL company the need to characterize the new resulting fractions and value the new by-products. The objective of this work was to value the juice clarification by-products, producing a clean label and fiber-rich snack, in a circular economy rationale, where the secondary products are upcycled back into the food value chain, by creating another food product that includes those by-products. For the above to be possible, the laboratory conditions to produce pear fractions were optimized. After optimizing the puree centrifugation, using response surface methodology (RSM), and optimizing the turbid juice crossflow filtration, the different fractions were characterized in rheological, nutritional, and physical aspects. Comparison to the pulps revealed an increase in the viscosity of the pomace; an enriching effect on the fructose, glucose, and dietary fiber levels in the pomace, and maintenance of the vitamin C levels after centrifugation; and with no effect on the contents of total phenols during the filtration step. A thick pear snack was developed, incorporating retained fraction, inulin, and *Euglena gracilis* in the pomace, and optimized regarding its firmness and dietary fiber content. The snack characterization revealed an interesting total phenols content (which was maintained from the raw materials). Compared to the snack without microalgae and a commercial fruit snack, the pear snack with *E. gracilis* was well-accepted by the sensory panel, mainly in texture and appearance, and can be further improved in aroma and flavor. The snack without microalgae was the favorite among the three samples, in most sensory parameters, and never got the answer “I'm sure I wouldn't buy it.” Therefore, an innovative, clean label and plant-based snack was developed, in a circular economy rationale, which was relatively well-appreciated by the panel. This snack is rich in dietary fiber, having the possibility of presenting various nutritional claims, and the potential for easy sensory optimization.

## Introduction

Recently, the consumer's preferences are for honest foods, like “natural” and “free-from” products, slightly processed and with a shorter ingredient list, which should be easily identified and understood. This major trend (usually called “Clean label”) resulted in pressure on the food industry to shorten the ingredients list, even on those being considered safe by regulatory agencies (1–3).

“Clean label” food products, although without any legal definition, are generally positioned as slightly processed, organic, “natural” and free from artificial ingredients, having the fewest number of ingredients possible. The consumer should also easily recognize these ingredients since some additives are perceived as harmful to health (despite legal approval) and the products are considered highly processed when they have a difficult name to pronounce (1–3).

The clean label is a rising food trend, involving health, sustainability, and environmental factors, and is usually linked to plant-based products and related to the circular economy concept, which is multidisciplinary and highly targeted both by academia and the industry (1–3).

It is widely known that 1/3 of the world's produced food is not consumed and is lost every year, and a great part of it is related to food wastage (4). Large amounts of by-products are produced by the food industry and, specifically the fruit juices industry, wastes from 20 to 60% of the raw materials as by-products (peels, seeds, and pomaces), depending on the process. The circular economy is a response to the environmental current challenges (high residuals production, resources scarcity, population growth). This multidisciplinary concept is being highly targeted and is related to sustainability and the substitution of the “end” idea by the reduction, alternative reuse, recycling, and recovery of several materials resulting from the different phases of the food chain. Thus, the circular economy is one of the pointed solutions to invert the above-described scenario, to produce value-added compounds by extraction processes, other food products, functional flours, biodiesel, sustainable packages, among others, and upcycling food industry side streams (5–11).

The snack developed in this work also fits into two important trends: snacking and microalgae consumption, both perfectly compatible. Although with no official definition, *snacking* is growing in the last years, a trend that prevailed also during the COVID-19 pandemic. This category is usually characterized by low amounts of food consumed apart from main meals, conveniently packed to be taken on the go. Liquid/thick snacks are a growing subcategory, which is expected to rise at a compound annual growth rate of 8%, by 2025. These snacks contain typically fruit, usually with some pieces, generating a satiating sensation, and being an interesting complement to the daily fruit apport, when consumed in the right amounts and formulated to have a positive impact on health (12–15).

Microalgae are an alternative food, recommended by the Food and Agriculture Organization of the United Nations, completely aligned with sustainability, “green revolution,” and plant-based food trends. Its production does not compete with any food culture, can be performed in non-arable land with non-drinking water, by a sustainable and environmentally friendly process—high efficiency, high growth rates, CO_2_ fixation, O_2_ production, and among others. Particularly, *Euglena gracilis* is a microalga whose consumption was recently approved by EFSA [under the *Commission Implementing Regulation (EU) 2020/1820 of 2 December 2020*] for bars, yogurts and yogurt beverages, fruit/vegetable beverages, fruit-flavored drinks, meal replacement beverages, food supplements, and total diet replacements for weight control. *E. gracilis* contains interesting amounts of paramylon (20–70% dry mass), an insoluble polysaccharide (1–3 beta-glucan), classified as dietary fiber, with proven beneficial immunomodulatory effects (immunity function stimulation). The addition to food products of *E. gracilis* can have beneficial effects, despite some rheological and, mainly, sensory impacts that still need to be addressed (16–18).

WHO recommends a daily intake of 400 g of fruits and vegetables for more than 10 years by now. However, many European countries still have a low fruit consumption frequency. In Portugal, 12% of the population eats just one to three portions of fruit per week, and the average consumption levels are 7.0% below the recommended values (19, 20). Therefore, initiatives to increase fruit consumption in Europe, and particularly in Portugal, are needed. Along with fruit consumption, there is usually the intake of dietary fiber, a particularly important group of compounds for the human diet and health since it contributes to satiation, obesity control, digestive health, immunity response, intestinal mucosa integrity, and among others. Inulin, a soluble and prebiotic fructo-oligosaccharide naturally extracted from chicory, is included in this category. Although a small polymer, it can influence the rheology, nutritional and sensory properties of foods when added in sufficient amounts (21).

Rocha do Oeste Pear (*Pyrus communis L*.) is a Portuguese Protected Designation of Origin variety of pear. It is one of the most relevant fruits in Portugal, not only in terms of production area but also in economic and exportation terms (in 2019, 60% of the production was exported). It is characterized by its firm pulp, high digestibility, interesting content in dietary fiber [2.3% (w/w)], and high amounts of ferulic acid, in comparison with other varieties (22, 23). It is also one of the fruits widely processed and studied by SUMOL+COMPAL, an important Portuguese fruit juice company that showed an obvious interest in the knowledge and valorization of by-products resulting from the production of pear juices, in particular pomaces resulting from juice clarification steps. The production of juices at the SUMOL+COMPAL pilot plant encompasses firstly puree production, followed by the centrifugation of pulps, to produce turbid juice, and by a crossflow filtration, to produce clarified juice (ultrafiltration). Pulps centrifugation allows the removal of fibers, mainly pectins and other insoluble carbohydrates, which are concentrated in the pomace (24). It is a process where the denser the particle is, the faster its settling occurs. Mainly the friction and buoyancy forces counteract the centrifugal force (25). Turbid juice crossflow ultrafiltration with membranes is a less costly alternative to several traditional methods (energy, labor, and capital), which prevents clarifying agents' usage, and avoids degradation of certain compounds and sensory characteristics. In fruit juices, this process changes the composition of the resulting juice by removing mainly polysaccharides and macromolecules, which are retained in the second by-product of the process (the retained fraction), together with some bioactive compounds (eventually). The main process disadvantage is the inevitable membrane fouling—which is dependent on several factors but can be delayed by crossflow and backflush usage—and the concentration polarization (26–31).

This work aimed to respond to the above-stated challenges and trends by developing and characterizing a thick and clean label fruit snack, incorporating *Euglena gracilis*, valorizing “Rocha” pear fruit juice by-products (also characterized), in a circular economy rationale. The production of turbid and clarified juice was optimized and characterized by mimicking, at a laboratory scale, the procedures done by the company at a pilot scale. This is one of the few studies focusing on the development of clean label fruit snacks, incorporating a microalga, and one of the few studies on “Rocha” pear puree processing and by-product valorization and characterization.

## Materials and Methods

### Samples

“Rocha” pear puree was provided by SUMOL+COMPAL. Briefly, 2019 campaign pears from the *Oeste* Portuguese region were washed, milled, and crushed. Ascorbic acid was then added to avoid oxidation, and the puree was subjected to thermal shock at 110°C (to inactivate polyphenol oxidases). The resulting puree was cooled to 80°C, refined to remove seeds and peduncles, and packed under heat conditions (70–80°C) in plastic jerricans, and finally frozen at −18°C. This puree was kept at −18°C, until being used in the laboratory. This matrix was the basis for all the further processing.

The puree was centrifuged, resulting in pomace (by-product) and turbid juice, which was after filtrated, resulting in the second pomace (by-product—from now on, called *retained fraction*) and in clarified juice. All the obtained fractions were kept at −18°C.

### Juice Production Processes Optimization

#### Puree Centrifugation Optimization

Turbid juice production by centrifugation was optimized using the Response Surface Methodology (RSM), set up for two independent variables—time (s) and spin rate (*xg*)—with a central composite rotatory experimental design at five levels (12 experimental conditions) (32), at a fixed temperature of 40°C. A *5810/5810 R* centrifuge (Eppendorf, Hamburg, Germany), equipped with the *F-34-6-38* rotor was used to perform these experiments. The dependent variable considered was the turbid juice Suspended Solids percentage (% SS, w/w), adapting the gravimetric method presented by Dahdouh et al. (33). RSM time ranged from 2 to 18 min, and spin rate from 300 to 3,000 *xg*, where the central point was measured in quadruplicate.

After performing the 12 assays correspondent to the 12 experimental conditions (in triplicates), the results were treated using *STATISTICA v10.0* software, and the predictive model that describes the relationship between spin time and rate and % SS (w/w) was obtained. The generic mathematical expression is given by the equation below (Equation 1):


(1) $$\begin{array}{l} \text{Y}=\beta_{0}+\beta_{1}*{\text{X}}_{1}+\beta_{2}*{\text{X}}_{2}+\beta_{12}*{\text{X}}_{1}*{\text{X}}_{2}\\ \;+\beta_{11}*{\text{X}}_{12}+\beta_{22}*{\text{X}}_{22} \end{array}$$

where Y is the % SS (w/w), β_0_ the compensation term, β_1_ and β_2_ are the linear effects, β_11_ and β_22_ the quadratic effects, and β_12_ the interaction effects between spin speed and time.

#### Turbid Juice Crossflow Filtration Optimization

Two 5 L turbid pear juice batches previously produced under the previously settled puree centrifugation conditions were used to optimize crossflow filtration. A *Pall*^®^ *Membralox*^®^ *XLAB 5 Benchtop crossflow pilot unit* (Pall Corporation, NY, USA) with a *Membralox T1-70 module* was used to filtrate. Two mono channel zirconia + alumina asymmetric ceramic membranes, with 50 (M50) and 100 nm (M100) pore size, were used. Turbid juice was fed at two different initial temperatures (50 and 60°C), in line with SUMOL+COMPAL processing, comprising a total of four different conditions (two initial temperatures for each membrane). Turbid juice was pre-heated in the jerrican to the initial temperature of filtration, in a water bath. The filtration occurred for 5 h, without temperature maintenance, with liquid recirculation [with a crossflow velocity of 5 m s^−1^ (1 bar)], using air backflush (4 bar), and periodically measuring the flow at 1.2 bar (time to reach 40 mL), in this case without air backflush.

Both turbid juice and the resulting fractions (retained fraction and clarified juice) were analyzed regarding turbidity, using the method described by O'Dell (34). Clarified juice yield [% (v/v)] was also calculated, at the end of each batch. At the end of each filtration, the system was washed, using the procedures given by *Pall Corporation* company and Pérez-Gálvez et al. (35). To confirm the effectiveness of the washing process, the flow was measured at the beginning of each filtration and the end of each washing process, using deionized water (25–27°C).

### Development of a Satiating and Clean Label Snack, Incorporating “Rocha” Pear Pomace and Retained Fraction, Chicory Inulin, and *Euglena gracilis* Microalga

To develop the clean label snack, a benchmarking analysis was initially performed, using six commercial fruit thick pouches/snacks and measuring the pH, color, and texture (using an acrylic probe with 19 mm diameter). Secondly, different formulations strictly with pomace and retained fraction were analyzed for both viscosity and firmness, aiming to reach a firmness within the range obtained in the benchmarking (target). Chicory inulin and pasteurization influences on the retained fraction viscosity were also analyzed in a controlled stress rheometer, using the CC25 DIN Ti probe and a thermostat heating water bath to pasteurize, at the levels proposed by Petruzzi et al. (36) for fruit juices.

The effects of chicory inulin and *E. gracilis* microalga addition and pasteurization in the pomace + retained fraction formulations were studied regarding viscosity (PP60 probe) and texture (19 mm diameter acrylic probe), using the pomace/retained fraction optimal ratio previously obtained. Inulin was added in a sufficient amount to reach 4 g of dietary fiber per 100 g of snack, allowing the European Union (EU) nutritional claim *Source of Fiber*, and *E. gracilis* was added, using the maximum amount authorized by the Commission Implementing Regulation (EU) 2020/1820 of 2 December 2020, for fruit and vegetable juices, nectars, fruit/vegetable blend beverages: 0.12% (w/w). All formulations were produced at 20°C, in a temperature-controlled room, by the following order: inulin and retained fraction mixing—*E. gracilis* addition and mixing—pomace addition and vigorous homogenizing—filling of glass recipients—pasteurization.

#### Pasteurization

Pasteurization was performed according to Petruzzi et al. (36) indications, to fruit juices with a pH higher than 4.5—High-Temperature-Long-Time pasteurization at 80°C/90 s to obtain, at least, a resistant microorganisms' reduction of 5 log, also inactivating any enzymes that have resisted to prior processing.

### Physicochemical Quality Parameters

The several fractions and formulations, as well as the final product, were analyzed by their total soluble solids (TSS - °Brix), color, pH, dry matter, and turbidity.

Total soluble solids (%) were evaluated by refractometry with a digital refractometer (*Atago PAL-1*- Japan), after removing suspended solids by a brief filtration (using a macroporous filter), except for clarified pear juice (practically without any suspended solids).

The color was measured by the *Chroma Meter CR-400* chromameter (Konica Minolta, Japan). The results were obtained in CIELab color space (L^*^, a^*^, b^*^), using a white tile (Y = 86.7; x = 0.3160; y = 0.3233) as a reference. Essays were performed until five similar points were obtained. Total color difference (ΔE) between two samples represents the degree of general color differences and was determined according to the equation below (Equation 2):


(2) $$\begin{array}{l} \Delta \text{E}=\sqrt[{2}]{{\left(L^{*}{\;}_{1}-L^{*}{\;}_{2}\right)}^{2}+{\left(a^{*}{\;}_{1}-a^{*}{\;}_{2}\right)}^{2}+{\left(b^{*}{\;}_{1}-b^{*}{\;}_{2}\right)}^{2}} \end{array}$$

where L^*^ (lightness), a^*^ (red-greenness), and b^*^ (blue-yellowness) are the obtained color parameters. Usually, color differences are detected by the normal human vision when ΔE > 5 (37).

Samples pH were analyzed using a *Hach 50 10T* electrode, associated with the *Basic 20* potentiometer (Hach Lange, Barcelona, Spain).

Dry matter was determined following the procedure described by Harris and Marshall (38), using 5 g of sample, in quintuplicates, drying at 105°C, until constant weight. Samples' dry matter was calculated using the equation below (Equation 3):


(3) $$\begin{array}{l} \text{Dry matter }\left(\text{\%}\right)=\frac{\text{Dry sample weight}}{\text{Humid sample weight}}*100 \end{array}$$

Finally, turbidity measurements were performed following the procedure described by O'Dell (34), using a *Hach 2100N IS Turbidimeter* (Hach Lange, Barcelona, Spain). When necessary, samples were diluted in previously boiled Mili-Q water, to minimize errors. The final result was calculated taking into account the used dilution ratio.

### Rheology Behavior Evaluation

#### Viscosity Evaluation

Viscosity curves were carried out using a rotational rheometer *Haake Mars Modular Advanced Rheometer System* (Thermo Fisher Scientific, Massachusets, USA) associated with the refrigeration system (Peltier) *Thermo Scientific Haake MarsIII Controller* and to the air compression system *Eheim professional 3*. The *PP60* probe (60 mm of diameter, rough surface) was used for the thick samples—puree, pomace, and snack/formulations—and the *CC25 DIN Ti* probe (concentric cylinders) was used for the other liquid samples. Viscosity curves were obtained in triplicate, with a 1.5 mm gap, at 20 ± 2°C, with shear rates ranging between 1 × 10^−8^ and 100 s^−1^, stepping up every 35 s to ensure the steady-state on each shear rate. Data treatment was carried out using *Origin 2019b (OriginLab)* software, adjusting the obtained curves to the *Cross* model (Equation 4):


(4) $$\begin{array}{l} \eta =\eta_{\infty }+\frac{\eta_{0}-\eta_{\infty }}{{1+\left(k*\overset{∙}{\gamma }\right)}^{m}} \end{array}$$

where η_∞_ (Pa.s) is the infinite shear viscosity, η_0_ (Pa.s) the zero-shear viscosity, k the consistency coefficient (Pa.s), m the *Cross* rate (dimensionless) constant, and ${\overset{∙}{\gamma }}^{.}$ The shear rate (s^−1^).

#### Texture Evaluation

Texture evaluation was carried out at 20 ± 2°C (in a temperature-controlled room), using a *TA.XT plus Texture Analyser* texturometer (Stable Micro Systems, Surrey, UK), equipped with a 5 kg load cell, using a 19 mm diameter acrylic probe. TPA tests were performed using 1 mm s^−1^ of speed, 30% penetration in the sample, 2 s between each penetration, which was placed in glass bottles (42.2 mm height and 43.5 mm diameter), till 30.7 mm height. Given the sample nature, in the end, only the maximum force peak (N) was used from the resulting force-distance curves, to characterize the several matrixes and optimize the texture of the snack. It is important to mention that these tests, performed in the snack development phase, were empirical. Although these measurements are more suitable for gels and more solid matrixes (39), this method was used and the justification for it is presented in the Discussion section.

### Nutritional Characterization

Samples nutritional analyses were carried out. Ash content was determined using the standard method (38), using the previously dried samples obtained from dry matter determination, and a *13/1300* muffle (Snol, Lithuania), at 550°C, overnight.

Crude protein was quantified by the Dumas method, based on the combustion of the sample in an oxygen-enriched atmosphere, at a high temperature, to ensure complete combustion. These determinations were done using the *NDA 702 DUMAS Nitrogen Analyser* (VELP Scientifica, Usmate Velate MB, Italy), equipped with a *VCopper* universal reductor and a TCD detector. 100 mg of sample were used, adding circa 50 mg of *VELP* absorbent powder. The determinations were made in triplicates. The result was expressed in protein grams per 100 grams of sample, using the 6.25 conversion factor for the obtained N_2_ amount (40, 41).

Lipid content was estimated using the *Soxtec* method described by Ijarotimi et al. (42), for the thick samples (puree and pomace). A quantity of 8.5 g of sample was dried (105°C, 24 h), to remove most of the water content. The weighted dried sample was added of 2.3 g anhydrous sulfate (Sigma-Aldrich Chemical Company, St Louis, MO, USA), in a thimble, and the extraction was performed, using the *Soxtec System HT 1043* extractor unit (Tecator, Hoganas, Sweden), and 40 ml of petroleum ether as solvent (b.p. 40–60°C, Fisher Scientific). Extracted fat was finally dried (105°C, 1 h), cooled, and weighted.

Total, soluble and insoluble dietary fiber determination was performed under the AOAC 991.43 official method, following the described steps by Lee et al. (43), using the Megazyme assay kit (K-TDFR-100A/K-TDFR-200A 04/17). Briefly, 1 g of dried pear samples (six replicates) were subjected to sequential enzymatic digestions by heat-stable α-amylase, protease, and amyloglucosidase. Soluble dietary fiber was obtained by chemical precipitation with EtOH and insoluble dietary fiber was obtained by filtration. After washing and drying, both fractions were weighted and the soluble, insoluble and total fiber contents calculated, using the Megazyme kit steps.

Reducing sugars were determined using the High-Performance Liquid Chromatography. 700.00 mg of sample were diluted with deionized water, in a 1:2 ratio. This mixture was centrifuged (16,000 × *g*, 5 min) and the supernatant was added of sulfuric acid 50 mM (Sigma, St. Louis, MO, USA), in a 1:10 ratio. The mixture was again centrifugated (in the same conditions) and the supernatant was finally filtered in disposable nylon filters of 0.2 μm pore diameter (Millipore, Cork, Ireland). 20 μL of the prepared samples were injected at 65°C, in duplicates, through an HPLC system, equipped with a Refraction Index Detector (*Refractive Index Detector 2414*- Waters Massachusetts, USA) and an Ionic Exclusion Column to sugar and organic acids analysis [*Rezex*™ *ROA Organic Acid H*+ *(8%) column, 300* × *7.8 mm*—Phenomenex, Torrance, CA, USA]. Sulfuric acid (5 mmol L^−1^, Sigma, St. Louis, MO, USA) was used as the mobile phase, and the used flux was 0.5 mL min^−1^. HPLC standard calibration curves for glucose and fructose were built by the injection of 13 different standard solutions, containing analytes at an increasing concentration ranging from about 0–36 g L^−1^ (glucose) and 0–42 g L^−1^ (fructose).

The total starch in the fractions was determined enzymatically according to the method described by Goñi et al. (44), David Barine and Yorte (45), and Reshmi et al. (46). The ground sample (100 mg) was dispersed in 6 ml of 2 M KOH and shaken at 4°C temperature for 30 min. Then, 3 ml of 0.1 M Sodium acetate buffer pH 4.75 and 60 ml of amyloglucosidase (EC-3.2.1.3, Sigma-Aldrich Chemical Company, St Louis, MO, USA) were added to this suspension and incubated for 45 min at 60 min at 60°C in a controlled shaking water bath. Then, 100 μl of the centrifuged solution (10,000 rpm, 15 min) was added of 400 μl of deionized water, 750 μl of DNS reagent, and, after 5 min at 100°C, 1,750 μl of deionized water. The solution absorbance was read, at 540 nm, in the *Cary 4000 UV-Vis spectrophotometer* (Agilent Technologies, Santa Clara, CA, USA), against a DNS and deionized water blank. A glucose calibration was prepared, with concentrations ranging from 0.1 to 2 mg ml^−1^. The conversion factor from glucose to starch was 0.9.

Titrable acidity was determined for the puree and the snack, using the method described by Pedro et al. (22), specifically for “Rocha” pear. Approximately 10 g of sample were diluted with deionized water, in a 1:5 ratio. The titration with 0.1 M NaOH (Sigma-Aldrich Chemical Company, St Louis, MO, USA) was controlled using a *Hach 50 10T* electrode, associated with the *Basic 20* potentiometer (Hach Lange, Barcelona, Spain), until a pH of 8.15. The result was expressed as malic acid equivalents (g 100 ml^−1^).

Antioxidant activity was measured both by DPPH radical scavenging and FRAP assays, since it is recommendable to use at least two different assays, given the sample and the method's possible variability (47, 48). For both methods, the samples (in triplicate) were extracted during 1 h at room temperature, using methanol:water solution (80:20), in a ratio of 1:2. After 1 h, the samples were centrifuged at 10,000 rpm, for 10 min. DPPH method was performed following the method described by Swain and Hillis (49) and Bunzel and Schendel (50). Diluted extract replicates volumes (1:2 ratio, with methanol:water 80:20) were mixed with a 103.5 μM DPPH solution (Aldrich Chemical Company, Milwaukee, WI, USA) and, after 60 min of incubation, the results (to insert in the calibration curve) were calculated in % RSA (percent radical scavenging activity) (Equation 5):


(5) $$\begin{array}{l} \text{\% RSA}=\frac{\text{AD}-\text{AS}}{\text{AD}}*100 \end{array}$$

where AD is the absorbance value at 517 nm of the DPPH methanolic solution and AS is the absorbance value at 517 nm of the sample solution.

FRAP spectrophotometric assay was performed using the method described by Benzie and Strain (51) and Rufino et al. (52). Diluted extract replicates volumes (1:6 ratio, with methanol:water 80:20) were mixed with a FRAP solution and, after 30 min of incubation at 37°C, the absorbances of the samples were measured. For both methods, control samples for each replicate were prepared without adding any pear extract, and the antioxidant activity was measured using the *Cary Series UV-Vis spectrophotometer* (Agilent Technologies, Santa Clara, CA, USA), at 517 nm (methanol as blank) and 595 nm (deionized water as blank), for DPPH and FRAP, respectively. The results were expressed as Trolox equivalents (mg TE 100 g^−1^), and the Trolox standard, for both calibration curves (Trolox concentrations ranging between 0 and 1,000 μM), was purchased from Aldrich Chemical Company (Milwaukee, WI, USA).

To measure total phenols, the same extraction process used in the antioxidant activity determination was performed. The total phenols were determined according to the Folin-Ciocalteu colorimetric method, described by Bunzel and Schendel (50). 150 μl of extract were added to 140 μl of Folin-Ciocalteu reagent and 2,400 μl of deionized water. Control samples were also prepared without adding any pear extract, for each replicate. After 2 h of incubation at room temperature, the total phenols were determined using the *Cary Series UV-Vis spectrophotometer* (Agilent Technologies, Santa Clara, CA, USA), at 725 nm (blank was prepared in the same way that samples, replacing the extract for pure methanol). The results were expressed as gallic acid equivalents (mg GAE 100 g^−1^). The Folin-Ciocalteu reagent was purchased from Panreac AppliChem (Barcelona, Spain), and the gallic acid standard was purchased from Sigma-Aldrich Chemical Company (St. Louis, MO, USA).

To evaluate ascorbic acid content, the AOAC 967.21 method (2,6-dichloroindophenol titrimetric method), optimized by Oliveira, Godoy and Prado (53), was used, since it is considered the official method of analysis for vitamin C. In this method, the vitamin C in pear fractions/product was determined by oxidizing it (in acid medium) with 2,6-dichloroindophenol (DCPIP) (Sigma-Aldrich Chemical Company, St Louis, MO, USA) to dehydroascorbic acid. The samples were diluted with deionized water, at a ratio of 1:4, and titrated with a 4 mg mL^−1^ DCPIP solution, detecting visually the equilibrium point (first pink color). The amount of ascorbic acid was determined, in triplicate for each sample, by using a calibration curve with ascorbic acid (Sigma-Aldrich Chemical Company, St Louis, MO, USA) solutions, with concentrations ranging from 0.0234 to 0.199 mg ml^−1^. The results were expressed as the mg of ascorbic acid per 100 ml of the sample.

The mineral composition was determined by Inductively Coupled Plasma Optical Emission Spectrometry (ICP-OES), following and adapting the method described by Yeung et al. (54). Firstly, 0.5 g of sample were digested using 12 ml of “royal water” (chloridric acid 37 % (v/v) (Chem-Lab, Zedelgem, Belgium) and nitric acid 65% (v/v) (Sigma-Aldrich Chemical Company, St Louis, MO, USA), at 105°C in a *DigiPrep MS* digestor (SCP Science, Quebec, Canada). The digested mixture was added of demineralized water until 50 ml of total volume. After sedimentation, the clarified was recovered and the ICP-OES analysis was performed (*iCAP 7000 series*—Thermo Fisher Scientific, Massachusetts, USA).

### Sensory Analysis

Sensory analysis was performed to assess the developed snack acceptability, to understand the sensory effect of adding *E. gracilis*, and to compare it with a commercial product of the same class (*Compal à colher de pera e ananás*). One untrained panel of 51 individuals was chosen (mainly students and researchers). A hedonic sensory test was performed, using three samples: a commercial pear thick snack, the developed thick snack, and the same formulation, without the microalgae. The individuals were asked to give information about their snack consumption habits before the evaluation took place. The evaluation was focused on the samples' general aspect, aroma, flavor, and texture, using a five levels scale, like Moskowitz et al. (55) suggested earlier. Additionally, individuals were asked to do a global evaluation of the samples, to express their purchase intention (also using a 5-levels scale), and to order the three samples by their preference. The three samples were randomly distributed to the 51 individuals, in recipients with about 5 g of sample, and randomly identified with letters and numbers. Between samples, a mouth wash with water was asked. The analysis took place in controlled and comfortable conditions regarding light, temperature, noises, and odors, in the sensory evaluation room, to prevent distractions and their influences on the analysis, as suggested by Nielsen (56). This sensory analysis is in accordance with data protection legislation and participants gave their written consent when completing the analysis sheet.

### Statistical Analysis

Regarding centrifugation optimization procedures, using response surface methodology, *STATISTICA v. 10* software was used to obtain the response surfaces. Also, an ANOVA analysis was performed, to determine significant factors (α = 0.05). To adjust the obtained rheological data to the *Cross* model, in viscosity determinations, *Origin 2019b* (*OriginLab*) software was used. All the remaining data were analyzed using *Prism 5* (*GraphPad*) software, calculating the means and standard errors of the replicates. ANOVA was performed to determine significant differences between the means, using the *Tukey* test to compare more than two samples, and the *T*-test to compare only two samples, setting for both a significance level at *p* < 0.05.

## Results

### Production of Pear Juice and By-Products Fractions

#### “Rocha” Pear Puree Centrifugation Optimization

To obtain the turbid juice and pomace fractions (useful for the clean label snack development), the centrifugation of the pear puree was optimized by RSM. The dependent variable was % SS (w/w), and the two independent variables were considered: X_1_ - spin time (s) and X_2_ - spin rate (*xg*). Twelve experimental points were tested, according to the selected experimental design.

The 12 points tested showed that the % SS (w/w) ranged between 0.245 and 0.814 % (w/w). After the results were inserted into the software, the model was generated (Equation 6). The regression coefficient, *p*-value, as well as residual plots (data not shown) were used to evaluate the adequacy of the developed model and validate it. By the ANOVA analysis parameters, the linear and the quadratic effects of time were not significant (*p* > 0.05), but only the interaction factor between spin time and rate.


(6) $$\begin{array}{l} Y=0.98-5.00\times 10^{-4}X_{2}+7.40\times 10^{-8}X_{2}^{2}+1.50\times 10^{-8}X_{1}X_{2} \end{array}$$

Where X_1_ is spin time (s) and X_2_ is spin rate (× *g*).

ANOVA parameters: *R*^2^ = 0.964; *p*-value = 4.27 × 10^−9^.

**Figure 1:**
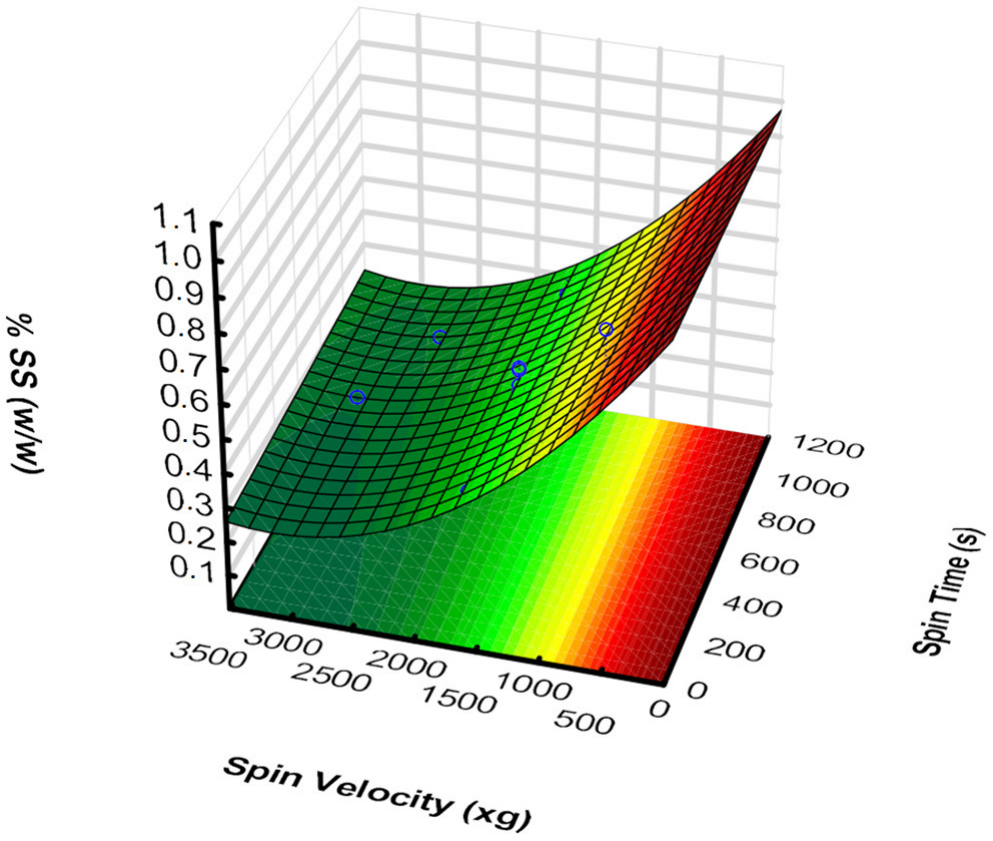
Response surface of the % SS (w/w) regarding spin time (X_1_) and rate (X_2_), for “Rocha” pear puree centrifugation.

The response surface was plotted (Figure 1), using the obtained model. Considering the obtained model, an optimal condition was established [270 s, 2,600 (× *g*)], to further produce turbid juice in higher amounts, taking into account the % SS (w/w), centrifugation time, and the approximated yield.

#### Turbid Juice Crossflow Filtration Optimization

Using two turbid juice batches produced in the previous “Rocha” pear puree centrifugation (270 s, 2,600 × *g*, 40°C), clarified juice production was optimized. Two turbid juice batches were used, each one for each pair of membrane tests.

**Figure 2:**
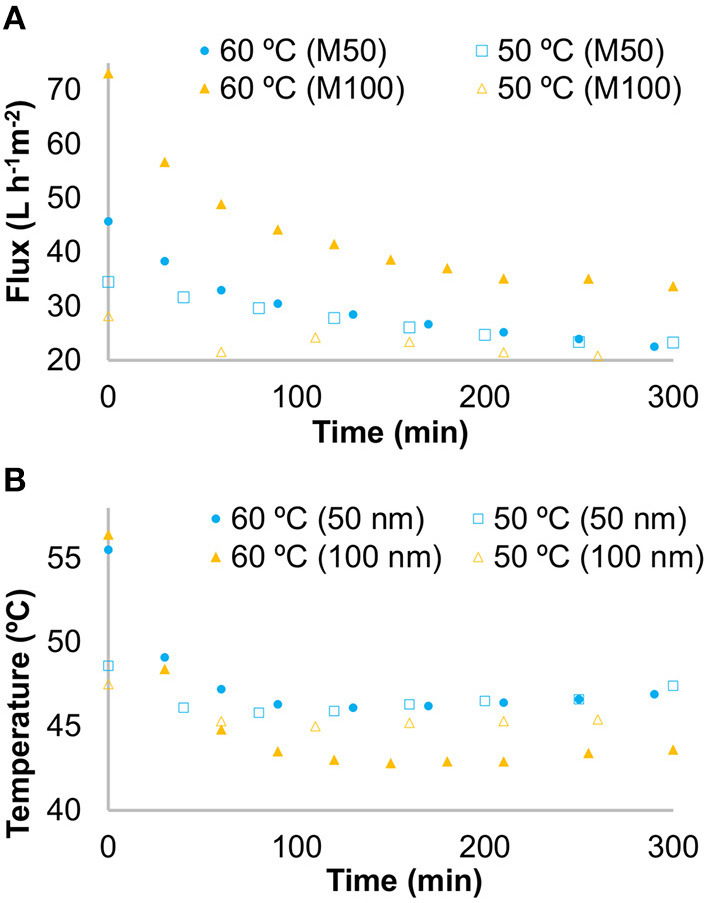
**(A)** Tangential filtration flux variation throughout the process (top graph). **(B)** Tangential filtration temperature variation throughout the process (bottom graph).

**Figure 3:**
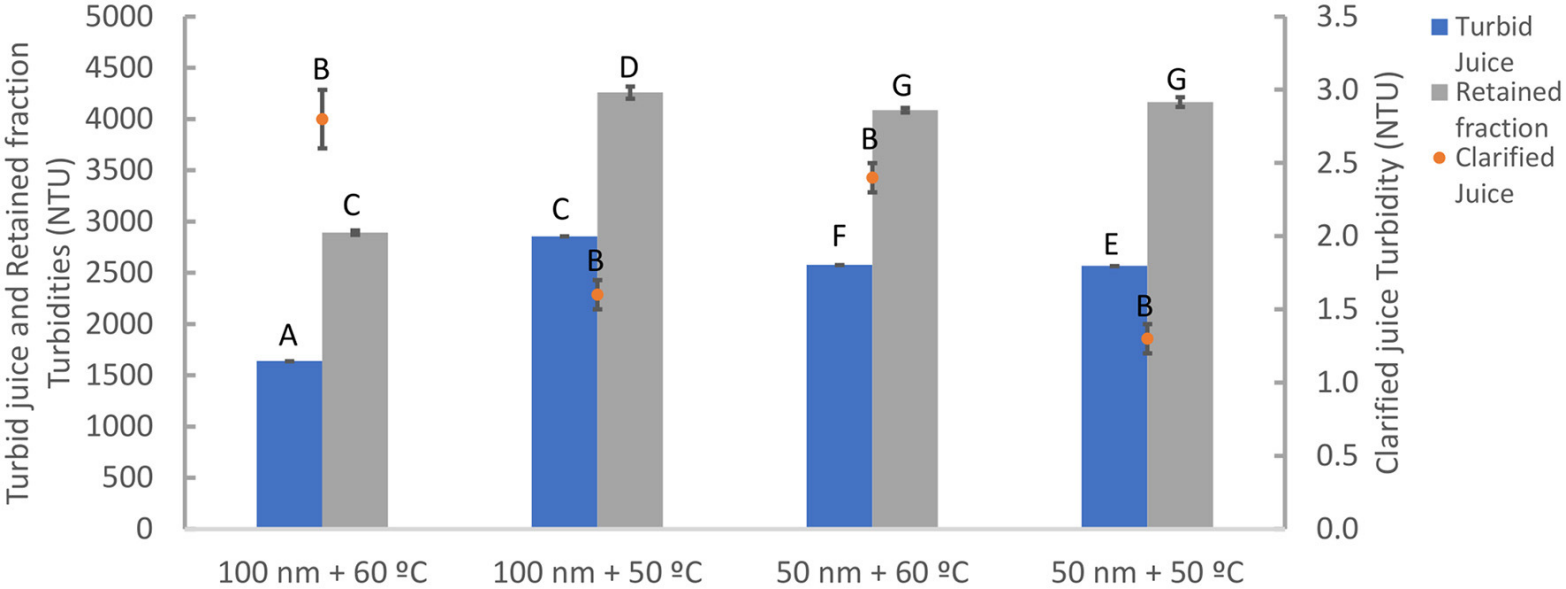
Measured turbidity values for the turbid and clarified juices, and for the retained fraction, in all filtration tests at 50 and 60°C of initial temperature, with M50 and M100. The letters presented are correspondent to the Tukey Test (α = 0.05).

The graphics in Figures 2A,B shows both fluxes and temperature variations, for the four tested. Figure 3 represents the turbidity for the turbid and clarified juices, as well as for the retained fractions in the several tested batches. From this figure, it can be observed that all the clarified juices do not significantly (*p* > 0.05) differ from each other. On the contrary, all turbid juices' turbidities differ significantly (*p* < 0.05), even more for the two M100 tests (at 50 and 60°C of initial temperature). However, both conditions tested with M100 showed that the clarified juice turbidity loss is similar (99.83% for 60°C and 99.95% for 50°C).

As it can be seen in Figure 2A, all the fluxes reduced gradually with time, tending to stabilize, which is in line with several filtration studies (35, 57). For the two M50 tests, the fluxes had similar behavior, starting higher at 60°C, but coinciding approximately after 75 min. For M100 tests, both fluxes differed, never being coincident, and the flux at an initial temperature of 60°C was always leading. Regarding temperatures (Figure 2B), it is clear that follows a similar behavior to that of the flux (a reduction with time), being the drop higher for the 60°C/M100 condition.

Total flow losses are higher for the conditions starting at 60°C, being similar for the same temperatures (in both membranes). The clarified juice yield was higher for M100/60°C (53.9%), the lowest yield was found in M100/50°C (30.4%), and the tests with M50 are similar in terms of this parameter (Table 1).

**Table 1:** Obtained clarified juice yields and total flow losses, for the four tested filtration conditions.

**Filtration condition**	**Turbid juice initial volume (mL)**	**Clarified juice yield (%)**	**Total flow loss (%)**
M 100; T_i_ = 60°C	2,000.0	40.0	53.9
M100; T_i_ = 50°C		27.5	30.4
M50; T_i_ = 60°C		35.0	50.7
M50; T_i_ = 50°C		36.0	32.6

### Pear Fractions Characterization

#### Nutritional Characterization

The pear fractions obtained and the puree were characterized in terms of nutritional composition, including the mineral profile (by ICP-OES), and the results are summarized in Tables 2, 3, on a wet basis.

**Table 2:** Nutritional and mineral characterization of “Rocha” pear fractions, on a wet basis (ND, not detected; “-”, not measured; <DL, below detection limit).

	**Humidity** ** (g)**	**Dry matter** ** (g)**	**Protein** ** (g)**	**Glucose** ** (g)**	**Fructose** ** (g)**	**Total lipids** ** (g)**	**Ash** ** (g)**	**Total dietary fiber** ** (g)**	**Insoluble dietary fiber** ** (g)**	**Soluble dietary fiber** ** (g)**	**Total phenols** ** (mg)**	**Antioxidant capacity (DPPH)** ** (μmol TE g^**−1**^)**	**Antioxidant capacity (FRAP)** ** (μmol TE g^**−1**^)**	**Ascorbic acid** ** (g)**	**Total starch** ** (g)**
	**Per 100 g of fresh matter**														
Puree	86.86 ± 0.095^a^	13.14 ± 0.095^f^	1.40 ± 0.046^k^	1.70 ± 0.004^l^	8.54 ± 0.012°	0.04 ± 0.012^r^	0.28 ± 0.040^s^	1.75 ± 0.19^v^	1.74 ± 0.18^x^	<DL	33.82 ± 1.824^C^	3.06 ± 0.317^E^	7.30 ± 0.309^G^	0.03 ± 0.003^L^	0.71 ± 0.228^N^
Pomace	85.21 ± 0.137^b^	14.79 ± 0.137^g^	1.46 ± 0.036^k^	1.83 ± 0.026^m^	9.43 ± 0.140^p^	0.02 ± 0.011^r^	0.19 ± 0.032^st^	3.52 ± 0.10^w^	3.43 ± 0.11y	0.18 ± 0.08^z^	29.35 ± 0.588^D^	3.62 ± 0.012^F^	8.92 ± 0.498^H^	0.03 ± 0.001^M^	0.66 ± 0.056^N^
Turbid juice	90.28 ± 0.061^c^	9.72 ± 0.061h	1.30 ± 0.050^k^	-	-	-	0.09 ± 0.065^tu^	<DL	<DL	<DL	29.63 ± 0.361^D^	3.55 ± 0.030^F^	7.39 ± 0.453^G^	0.03 ± 0.000^L^	0.56 ± 0.169N
Retained fraction	87.94 ± 0.005^d^	12.05 ± 0.005^i^	1.29 ± 0.031^k^	1.83 ± 0.003^m^	9.18 ± 0.017^p^	-	0.17 ± 0.056^t^	<DL	<DL	<DL	28.26 ± 1.445^D^	3.60 ± 0.032^F^	6.55 ± 0.407^I^	ND	0.65 ± 0.281^N^
Clarified juice	89.24 ± 0.020^e^	10.75 ± 0.020^j^	1.20 ± 0.081^k^	1.59 ± 0.007^n^	7.99 ± 0.037^q^	-	0.26 ± 0.044^u^	ND	ND	ND	27.78 ± 2.172^D^	3.56 ± 0.071^F^	5.71 ± 0.551^J^	ND	-

*The values presented in this table are means of triplicates ± standard deviation, with the letter corresponding to the Tukey test (α = 0.05) in the exponent*.

**Table 3:** Mineral characterization of “Rocha” pear fractions, on a wet basis.

	**Sodium** ** (mg)**	**Potassium** ** (mg)**	**Calcium** ** (mg)**	**Magnesium** ** (mg)**	**Phosphorous** ** (mg)**	**Sulfur** ** (mg)**	**Iron** ** (mg)**	**Copper** ** (mg)**	**Zinc** ** (mg)**	**Manganese** ** (mg)**	**Boron** ** (mg)**
	**Per 100 g of fresh matter**										
Puree	5.84 ± 0.972^a^	128.88 ± 2.762^c^	9.24 ± 0.479^e^	6.85 ± 0.179^h^	11.61 ± 0.434^k^	7.91 ± 0.385^mn^	2.51 ± 1.076°	0.21 ± 0.044^p^	0.16 ± 0.002^q^	0.06 ± 0.002^r^	0.31 ± 0.008^s^
Pomace	4.33 ± 0.296^b^	135.89 ± 1.137^d^	11.04 ± 0.721^f^	8.05 ± 0.136^i^	12.50 ± 0.184^l^	8.33 ± 0.181^m^	3.12 ± 3.362°	0.183 ± 0.016^p^	0.17 ± 0.015^q^	0.06 ± 0,010^r^	0.32 ± 0,014s
Retained fraction	5.47 ± 0.033^ab^	129.77 ± 1.96^c^	5.28 ± 0.098^g^	5.81 ± 0.161^j^	11.94 ± 0.086^kl^	7.39 ± 0.137^n^	1.41 ± 0.693°	0.22 ± 0.010^p^	0.16 ± 0.004^q^	0.05 ± 0.002^r^	0.24 ± 0.012^t^

*The values presented in this table are means of triplicates ± standard deviation, with the letter corresponding to the Tukey test (α = 0.05) in the exponent*.

The results show no significant (*p* > 0.05) differences between the puree and the pomace, and that they are comparable to other “Rocha” pear studies, both in terms of puree moisture and ash content (58, 59), and protein and total starch (22, 60). In terms of total lipids, there are no significant (*p* > 0.05) differences between the puree and the pomace, being the obtained values close to Pedro et al. (22) studies.

In terms of reducing sugars, fructose values for puree (8.54 ± 0.012 g 100 g^−1^) are considerably higher than those reported in the literature for this variety (61). On the other hand, glucose values (1.70 ± 0.004 g 100 g^−1^) are similar. Regarding the produced fractions, it is visible that the concentrated fractions are similar, being richer, and the clarified juice is the fraction poorest in sugars.

Regarding total dietary fiber, puree values (1.75 ± 0.19 g 100 g^−1^) are within the values obtained by Pedro et al. (22) and Soares et al. (58), for “Rocha” pear. For the other fractions, it is clear that the pomace fraction shows the highest (*p* < 0.05) content of total and insoluble dietary fiber and that turbid and clarified juices and retained fraction have residual amounts of dietary fiber, standing out its non-detection in the clarified juice.

In what concerns the antioxidant capacity, pear puree measurement (3.06 ± 0.317 μmol TE g^−1^) was within the range founded by Kolniak-Ostek (62) and Liaudanskas et al. (63) for DPPH assay, and in agreement to Ruiz-Torralba et al. (64) results, for FRAP assay (7.30 ± 0.309 μmol TE g^−1^), for different pear varieties. Regarding the other fractions, and focusing DPPH assay, a significant (*p* < 0.05) increase in the activity for the puree was observed. Focusing on FRAP assay results, it is noticeable that the pomace showed the highest (*p* < 0.05) activity (8.92 ± 0.496 μmol TE g^−1^) and that there is a reduction of the antioxidant activity with crossflow filtration, mainly in the clarified juice (5.71 ± 0.551 μmol TE g^−1^).

In terms of total phenolic compounds, no significant (*p* > 0.05) differences were detected between pomace, turbid juice, clarified juice, and retained fraction. The obtained value for the puree (33.82 ± 1.824 mg 100 g^−1^) is lower than the value reported by Önal et al. (65) to “Rocha” pear. About the remaining fractions, there is a significant (*p* < 0.05) loss in total phenols, in the centrifugation. However, the value obtained for the turbid juice (29.63 ± 0.361 mg 100 g^−1^) is higher than the obtained by Rocha et al. (66) for pear juices produced by centrifugation. All total phenols content, for the three liquid fractions, are contained in the range obtained by Tarinoven and Eski (67), for different pear cultivars' juices.

Focusing ascorbic acid analyses, the obtained puree value (0.03 ± 0.003 g 100 g^−1^) is above Pedro et al. (22) results. It is also noticeable that the centrifugation did not affect significantly (*p* > 0.05) the contents in the resulting fractions when compared with the puree. However, the crossflow filtration step reduced significantly (*p* < 0.05) the ascorbic acid content of both retained fraction and clarified juice (both non-detectable), in line with the reduction in the antioxidant activity observed.

In terms of mineral profile (Table 3), it was observed that the most abundant mineral on the puree, pomace, and retained fraction was potassium, followed by phosphorous, as in Coelho et al. (68). For most minerals, pomace presents the highest values, as expected, since it is the most concentrated fraction in solids. Also, in general, and for the three pear fractions, the obtained values are higher than the ones in the literature (68, 69).

#### Physicochemical Analysis

Table 4 shows the results for Soluble Solids (°Brix), pH and Turbidity, regarding all pear fractions.

**Table 4:** Determined physicochemical parameters for all the “Rocha” pear fractions.

**Fraction**	**TSS (**°**Brix)**	**pH**	**Turbidity (NTU)**
Puree	12.5 ± 0.084^a^	4.60 ± 0.079^f^	7,010 ± 619.903^i^
Turbid juice	12.2 ± 0.100^c^	4.51 ± 0.010^f^	2,576 ± 46.019^k^
Pomace	12.9 ± 0.153^bd^	4.62 ± 0.033^f^	14,476 ± 239.537^l^
Retained fraction	12.7 ± 0.000^d^	4.46 ± 0.006^f^	4,087 ± 75.200^m^
Clarified juice	11.8 ± 0.058^e^	4.47 ± 0.006^f^	2.35 ± 0.091^n^

*The values presented in this table are means of triplicates ± standard deviation, with the letter corresponding to the Tukey test (α = 0.05) in the exponent*.

For the soluble solids results, almost all soluble solids values for all fractions are significantly different (*p* < 0.05), being the lowest values found on turbid and clarified juices (12.2 ± 0.100 °Brix and 11.8 ± 0.058 °Brix, respectively). The pear puree value (12.5 ± 0.084 °Brix) is in line with the “Protected Designation of Origin 'Rocha' pear Book of Specifications” range, as well as with the values measured earlier (22), for the same variety. It is also visible that the value obtained for the turbid juice is in line with previous (66) studies.

The obtained pH value for the puree fraction (4.60 ± 0.079) agrees with Pedro et al. (22) results for this pear variety. None of the remaining fractions have significantly (*p* > 0.05) different pH values between them and the puree.

Fractions turbidity comes from pectins and other cell wall components presence, in suspension. Markowski et al. (70) point 250 NTU as the minimum turbidity for turbid juices, obtaining an interval between 590 and 1677 NTU for those. Clearly, and as expected, all fractions significantly (p < 0.05) differ from each other. The pomace fraction shows the highest value among all (14,476 ± 239.537 NTU), followed by the puree (7,010 ± 619.903 NTU). The next higher value is shown by the retained fraction (4,087 ± 75.200 NTU), followed by the turbid juice (2,576 ± 46.019 NTU), and, finally, for the clarified juice (2.35 ± 0.091 NTU), the one who has significantly (*p* < 0.05) the lower turbidity. All fractions have their turbidities above the values obtained by Markowski et al. (70).

#### Apparent Viscosity

The results about fractions flow behavior (using the *Cross* model) are presented in Table 5. Puree and pomace show a similar behavior: for reduced shear rates, the viscosity is constant (η_0_), followed by a shear thinning behavior, where the viscosity continuous and gradually decreases with the shear rate growth. For the turbid juice and the retained fraction, the behavior is similar: first, a continuous and gradual reduction of the viscosity with shear rate growth and, finally, a constant viscosity area (η_∞_) for higher shear rates.

**Table 5:** Rheological parameters of the flow curves adjusted to the *Cross* equation, for the several pear fractions.

**Fraction**	**η_0_ (Pa.s)**	**η_∞_ (Pa.s)**	**m**	**k (s)**
Puree	3.06 × 10^4^ ± 7.527 × 10^3 a^	29.00 ± 50.249^c^	0.88 ± 0.202^d^	1.94 × 10^3^ ± 6.590 × 10^2^ ^f^
Pomace	2.32 × 10^5^ ± 3.038 × 10^4^ ^b^	2.81 × 10^−6^ ± 3.910 × 10^−6 *c*^	0.82 ± 0.047^d^	1.97 × 10^3^ ± 3.580 × 10^2^ ^f^
Turbid juice	84.58 ± 126.122^a^	0.06 ± 0.019^c^	1.20 ± 0.132^e^	7.50 × 10^5^ ± 1.091 × 10^6^ ^f^
Retained fraction	12.71 ± 7.613^a^	0.06 ± 0.003^c^	0.75 ± 0.053^de^	1.65 × 10^5^ ± 1.333 × 10^4^ ^f^

*The values presented in this table are means of triplicates ± standard deviation, with the letter corresponding to the Tukey test (α = 0.05) in the exponent*.

As it can be seen, the pomace is the fraction significantly (*p* < 0.05) more viscous, as expected, followed by the puree. Regarding liquid fractions, there are no significant (*p* > 0.05) changes in the infinite limiting shear viscosity values, even considering that the retained fraction has more suspended solids. Also, the zero-shear limiting viscosity, for the retained fraction and turbid juice, is below the obtained ones for puree and pomace.

Regarding k and m values, pear puree and pomace do not significantly (*p* > 0.05) differ from each other, as also happens for both retained fraction and the turbid juice. For all tested fractions, regression coefficients (*R*^2^) were always higher than 0.999, showing a good fit of the *Cross* model for the obtained data.

Given the very fluid characteristics of the clarified juice, with very low viscosity, it was not possible to obtain its flow curve with this equipment.

### Development of a Clean Label Snack, Incorporating “Rocha” Pear Fractions, and *E. gracilis*

The snack was designed and optimized having into account both its firmness (similar to other commercial fruit thick snacks) and its content in dietary fiber (enough to, at least, allow the EU nutritional claim *Source of Fiber* − 3 g of dietary fiber per 100 g of product or 1.5 g of dietary fiber per 100 kcal), aiming to incorporate *E. gracilis*, recently approved by EFSA for human consumption.

Firstly, a benchmarking analysis was performed on several commercial pear and/or apple thick snacks, regarding their consistency, color, and pH. Commercial samples' firmness (empirical testing) ranges from 0.122 N to 0.231 N. This is the target for the idealized snack, to be positioned in the same segment. Also, it was possible to measure that pomace has a higher (*p* < 0.05) firmness, in comparison with the commercial samples − 0.725 N. It was possible to define a pH range from 3.38 to 3.83, lower than the juice materials of this study, as well as the color range: L^*^ from 39.37 ± 0.37 to 49.12 ± 0.30; a^*^ from −0.03 ± 0.08 to 1.60 ± 0.07; and b^*^ from 3.38 ± 0.01 to 3.83 ± 0.01.

#### Basis-Formulation Optimization

**Figure 4:**
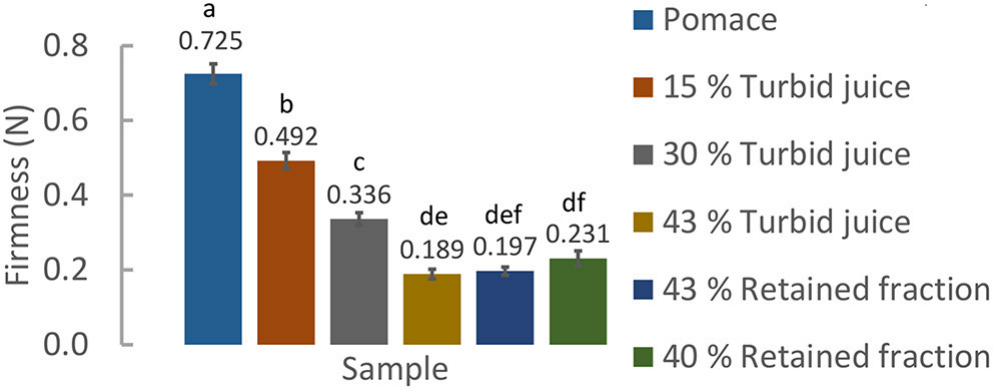
Obtained firmness (*N*) for the turbid juice/retained fraction and pomace formulations. The letters presented are correspondent to the Tukey test (α = 0.05).

Since the pomace firmness is significantly (*p* < 0.05) higher compared to the commercial samples, retained fraction (the other by-product) was added to reduce this value to the targeted firmness range. Given the low retained fraction amounts available, turbid juice was used in the first formulations. Therefore, 15, 30, 40, and 43% of juice/retained fraction incorporation into the pomace [% (m/m)] were tested. Figure 4 shows the firmness results of these formulations.

#### Chicory Inulin Powder Addition Effect on Retained Fraction Viscosity and Snack Firmness

Chicory inulin powder (with 89 % (w/w) of fiber) was added to, at least, meet the minimum legal amount of dietary fiber (3 g 100 g^−1^) to achieve the EU fiber claim. Reach 4 g 100 g^−1^, as a safety margin, was aimed, considering that: (i) puree centrifugation removes all soluble fiber from the puree as well as some insoluble one; (ii) retained fraction do not have dietary fiber; (iii) microalgae dietary fiber content is neglectable; (iv) yield of turbid juice is around 50% (w/w). The calculated amount of fiber in the pomace was 2.7% (w/w). Therefore, the inulin powder/pomace ratio should be 0.046 to reach 4 g of fiber per 100 g of snack.

**Figure 5:**
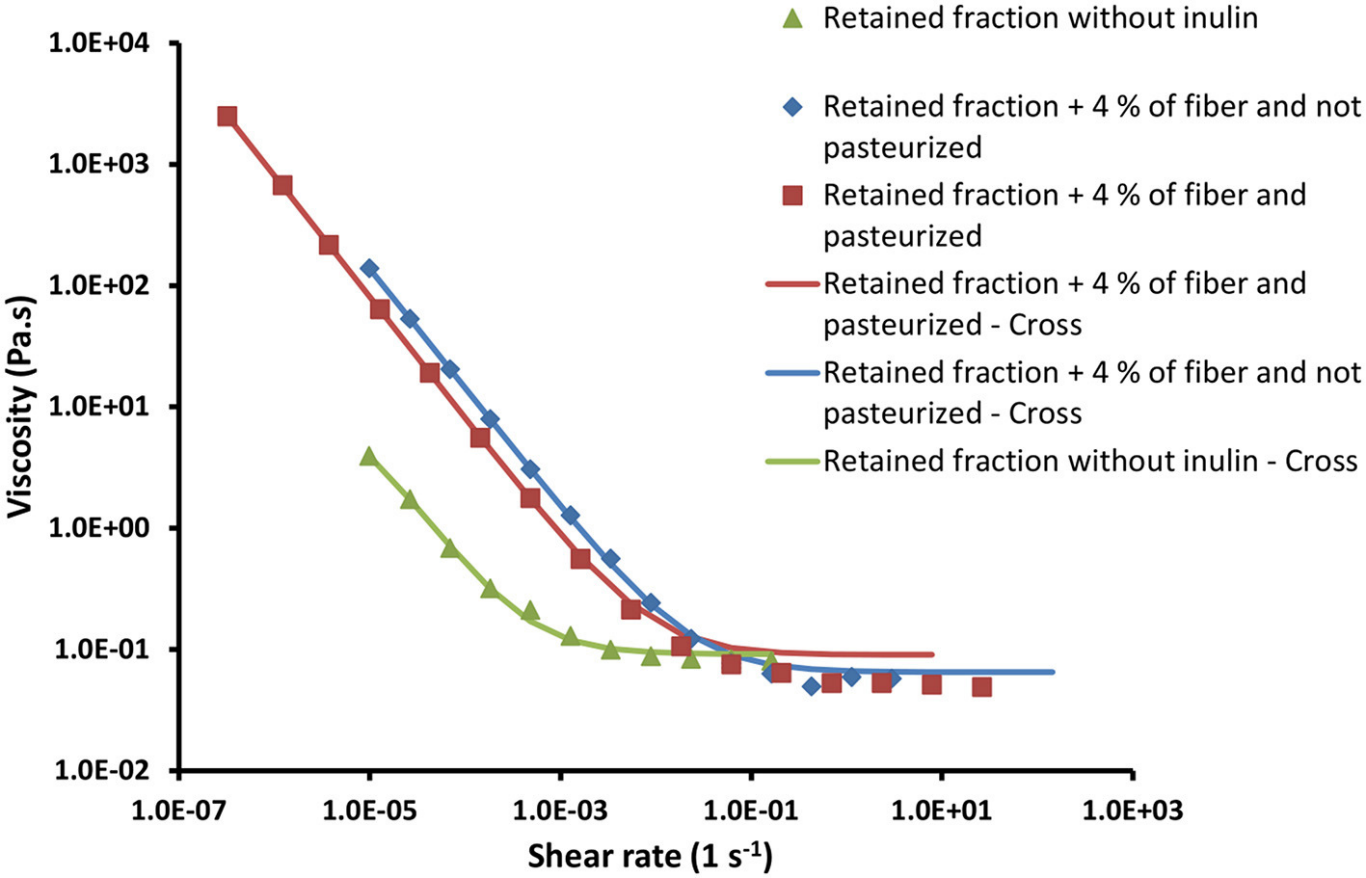
Flow curves of the retained fraction, the retained fraction with inulin, and the retained fraction with inulin and pasteurized.

To assess the rheological effect of the inulin, solutions of 4 g of fiber per 100 g of retained fraction (assuming no fiber on this fraction) were prepared. Flow curves were determined for retained fraction, the retained fraction with inulin, and this one pasteurized. None of the formulations showed significant (*p* > 0.05) differences on the limiting infinite viscosity zone. However, inulin addition had some impact on the shear thinning zone of the curves (Figure 5).

After assessing the inulin rheological effect on the retained fraction, which was neglectable, and considering the calculated fiber content of the pomace, inulin powder was added to the previously optimized formulation of pomace and retained fraction (to reach 4 g of fiber per 100 g), to again understand its impact on the firmness. Formulations (i) without inulin, (ii) with inulin and pasteurized, and (iii) pasteurized with inulin, measured after 3 days, were tested. The first formulation had a firmness of 0.231 ± 0.0200 N, the second 0.216 ± 0.0121 N, and the third 0.235 ± 0.0040 N. None of these values differ significantly (*p* > 0.05).

#### *Euglena gracilis* Incorporation

The last formulation step was to add *E. gracilis*, at 0.12% (w/w), the maximum content allowed by the Commission Implementing Regulation (EU) 2020/1820 of 2 December 2020, to *Fruit and vegetable juices, nectars, fruit/vegetable blend beverages*. The average firmness of the pasteurized formulation, with these microalgae, was 0.218 ± 0.0131 N [not significantly (*p* > 0.05) different from the formulations above mentioned].

Therefore, the final formulation includes [% (w/w)] 58.30% of pomace, 38.85% of retained fraction, 2.75% of inulin powder, and 0.12% of *E. gracilis*.

### Snack Characterization

After its development, the final snack was characterized regarding nutritional (Table 6) and physic-chemical composition.

**Table 6:** Nutritional characterization of the developed clean-label snack, with “Rocha” pear by-products, chicory inulin, and *E. gracilis*, in both wet and dry matters.

	**Humidity** ** (g)**	**Dry matter** ** (g)**	**Protein** ** (g)**	**Total lipids** ** (g)**	**Glucose** ** (g)**	**Fructose** ** (g)**	**Ash** ** (g)**	**Dietary fiber** ** (g)**	**Titrable acidity** ** (g of malic acid)**	**Total phenols** ** (g)**	**Antioxidant capacity (DPPH)** ** (μmol TE g^**−1**^)**	**Antioxidant capacity (FRAP)** ** (μmol TE g^**−1**^)**	**Ascorbic acid** ** (g)**	**Total starch** ** (g)**
Per 100 g of fresh matter	83.73 ± 0.043	16.27 ± 0.043	1.41 ± 0.047	0.02^*^	1.65 ± 0.000	8.52 ± 0.014	0.16 ± 0.084	4.51^*^	0.12 ± 0.003	0.03 ± 0.001	370.21 ± 124.250	620.16 ± 33.140	0.00 ± 0.000	0.64^*^
Per 100 g of dry matter	-	-	8.67 ± 0.286	0.12^*^	10.15 ± 0.000	52.37 ± 0.117	1.01 ± 0.518	27.72^*^	0.71 ± 0.016	0.18 ± 0.004	2,275.40 ± 95.204	3,811.69 ± 203.688	0.01 ± 0.001	3.93^*^

* *Means that the value is an estimation (it was not determined), giving the ratio of the different ingredients added*.

*The values presented in this table are means of triplicates ± standard deviation*.

#### Snack Physicochemical Characterization

The final snack presents a TSS of 15.15 ± 0.100 °Brix, significantly (*p* < 0.05) higher than all previous pear fractions. The measured pH value is 4.81 ± 0.037, also significantly (*p* < 0.05) superior to all pear fractions, as well as to the benchmarking obtained range of pH values.

Regarding color, an L^*^ of 52.15 ± 1.146, an a^*^ of −0.56 ± 0.207, and a b^*^ value of 15.52 ± 0.960 were obtained for the final snack (with microalgae), and an L^*^ of 58.51 ± 0.441, an a^*^ of 1.10 ± 0.23 and a b^*^ of 13.99 ± 0.174, for the formulation without microalgae. The total color difference between these two formulations is 7.04, visible to the human eye (37).

#### Snack Nutritional Characterization

The most important nutritional analysis results are pointed out below.

In Table 6 it is possible to observe that the water content of the snack is significantly (*p* < 0.05) lower compared with all the other pear fractions. Regarding total phenols, results show that the snack production (involving heat treatment and some atmosphere contact) does not affect significantly (*p* > 0.05) the final value, in comparison with pomace and retained fraction, and this was the same for DPPH results. These findings are in line with Dereli et al. (71) studies when pasteurizing carrot juices. Regarding the FRAP method, the result was not significantly (*p* > 0.05) different from the retained fraction, but it was from the pomace. Regarding the ascorbic acid results, it was impossible to determine it since the content was below the detection value.

Concerning dietary fiber content, a value that allows not only the *Source of Fiber*, but the *High in Fiber* nutritional claim is expected, and with a higher content, compared to all benchmarking products (it has almost the double content of the one found in the *Compal à Colher de Pera e Ananás* snack, for example).

About reducing sugars, fructose prevails as the main sugar, and both fructose and glucose contents aren't significantly (*p* > 0.05) different from puree contents, being significantly (*p* < 0.05) lower than pomace and retained fraction contents.

In terms of mineral composition (Table 3) and given the reduced amounts of added microalgae and inulin, it is expected that the profile remains approximately the one for the pomace and retained fraction.

It is expected that this snack contributes to 1 mg of paramylon per 100 g, an important bioactive to modulate the immune response, apported by the microalgae.

### Snack Sensory Analysis

To assess the acceptability of the developed clean label product by the end consumer, a sensory analysis was performed, involving three samples: the developed snack (with microalgae), a similar snack, but without the microalgae, and a commercial sample (similar texture to the developed one, also with “Rocha” pear, and also without *E. gracilis*). The panel of 51 individuals gave information about their snack consumption habits, sample sensory analysis, and, finally, their purchase intents and sample preference.

Panel ages were comprised between 18 and 82 years old (20–29 age group was predominant − 42.0% of the total), and the group had 58.9% of female individuals. Of the total of 51 individuals, 76.5% consume snacks often, 45.1% of which do it some days per week. 94.1% of the 51 individuals find interesting the microalgae presence in snacks, while 92.1% find important the existence of a higher variety of clean label snacks with natural ingredients.

**Figure 6:**
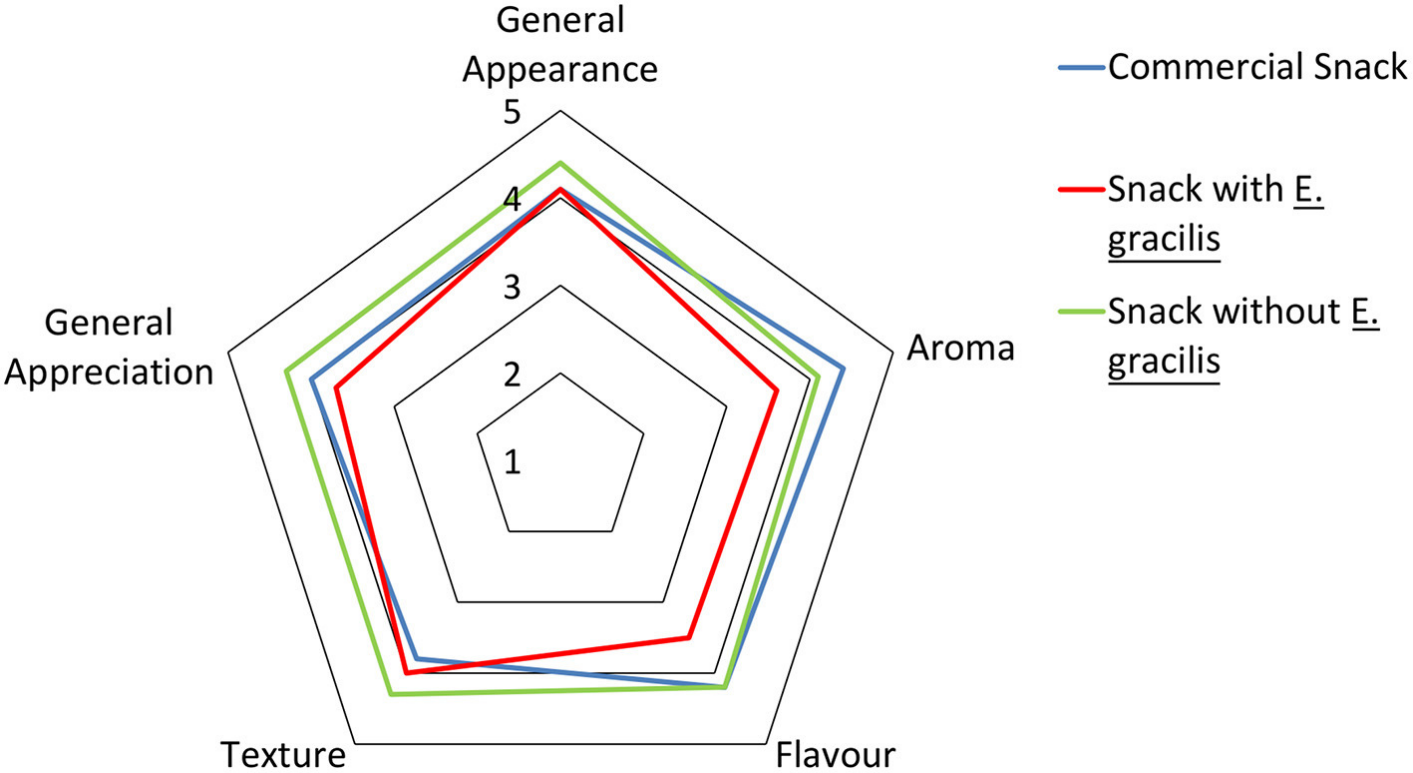
Radar chart, representing the classifications of the several sensory attributes obtained in the snacks' sensory analysis (*n* = 51). 1, Very unpleasant; 2, Unpleasant; 3, Indifferent; 4, Pleasant; 5, Very pleasant.

Figure 6 shows the sensory analysis results. It is possible to see that, in terms of general appearance, flavor (along with the commercial sample), texture, and general appreciation, the snack without microalgae is preferred. In terms of aroma, the winner is the commercial sample. Globally, the snack without microalgae and, next, the commercial snack are the most preferred samples. By the comments collected in the questionnaires, two individuals identified the microalgae flavor and aroma as positive aspects, however the overall negative feedback about the developed snack is related to this since most of the feedback mentions a “strange taste,” an “intense algae aroma,” when “a pear aroma was expected.”

In terms of the most preferred snack, the first sample was the commercial snack, with 49.02% of the individuals classifying it in the first place. Next, the snack without microalgae, also with 49.02% of the individuals evaluating this in second place. The least preferred sample was the snack with microalgae, where 52.94% of the individuals voted for third place.

**Figure 7:**
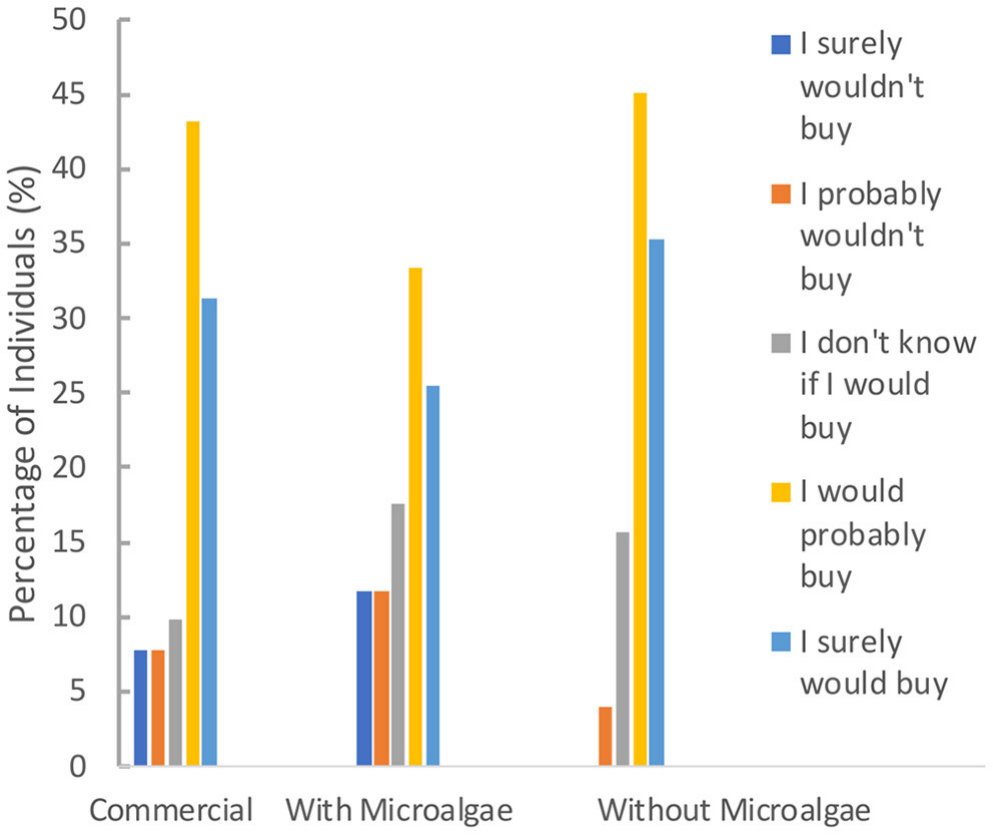
Purchase intention of the 51 individuals who participated in the snacks' sensory analysis.

Figure 7 presents the purchase intentions of the 51 individuals, regarding each sample. As it can be seen, for the three samples predominated both “I surely would buy” and “I would probably buy” − 74.5% for the commercial sample, 58.8% for the snack with microalgae, and 80.4% for the one without microalgae. The latter was the only one that never got “I'm sure I wouldn't buy” as an answer and, in general, overcame the other two. It was followed by the commercial sample and, finally, by the snack with microalgae, in agreement with the sensory results shown in Figure 6.

### Nutritional Claims on the Developed Snack

As previously mentioned, it is possible to apply some nutritional claims, considering the snack composition (Table 7), and both the Regulation (EC) No 1924/2006 of the European Parliament and of the Council of 20 December 2006 on nutrition and health claims made on foods and the Commission Implementing Regulation (EU) 2020/1820 of 2 December 2020 authorizing the placing on the market of dried *Euglena gracilis* as a novel food under Regulation (EU) 2015/2283 of the European Parliament and of the Council and amending Commission Implementing Regulation (EU) 2017/2470. The carbohydrates content estimation (starch, reducing sugars, saccharose, and sorbitol) was calculated having into account the nutritional results obtained in pomace and retained fraction analysis, as well as the reported values of saccharose in pear purees and pear juice and of the sorbitol content in “Rocha” pear purees (61, 66).

**Table 7:** Approximated snack nutritional composition regarding carbohydrates, protein, total lipids, dietary fiber, sodium, and energy.

**Ingredient**	**Proportion** ** (g per 100 g of *snack*)**	**Carbohydrates** ** (g)**	**Total lipids** ** (g)**	**Protein** ** (g)**	**Dietary fiber** ** (g)**	**Sodium** ** (g)**	**Energy** ** (kcal)**
Pomace	58.30	8.08	0.01	0.85	2.05	0.0025	38.59
Retained fraction	38.85	5.18	0.00	0.50	0.00	0.0021	7.96
Chicory inulin	2.75	0.22	0.00	0.00	2.45	0.0000	5.78
*E. gracilis*	0.12	0.08	0.01	0.02	0.01	0.0016	0.51
Total (g)	**100.00**	**13.56**	**0.02**	**1.37**	**4.51**	**0.0062**	**52.84**

## Discussion

### Production of Pear Juice and By-Products Fractions

#### “Rocha” Pear Puree Centrifugation Optimization

The obtained equation for the speed and time of centrifugation has a high significance, given the small *p*-value observed. Also, the model is very well-adjusted to the data, given its high regression coefficient value, near 1, explaining almost every observed variability. The residual vs. predicted values plot (data not shown) also supports the good fit of the predictive model on the experimental data as it exhibited random distribution patterns. Therefore, the model was validated.

The resulting surface (Figure 1) suggests that, as expected, a decrease in spin rate increases the % SS (w/w), since there is a fewer centrifugal force acting over the settling of the puree suspended solids. Given the obtained % SS (w/w) range, this can be an easy and fast way to produce a well-clarified turbid juice, although with low SS amounts, but leading to a potential application of the by-products, since the juice yields ranged between 8.04 and 71.43% (v/v)—the higher the yield, the smaller the % SS (w/w).

This model can be useful to predict the % SS (w/w), an important parameter that directly influences juices' dietary fiber content. This can be particularly important in the development of functional products, as a clean label snack. Depending on the objectives regarding, for example, process speed and % SS (w/w), several conditions can be optimized. The optimal condition 270 s, 2,600 (×*g*) was selected from the model, to produce, in the laboratory, turbid juice in higher amounts (with 0.25% (w/w) of SS). It was chosen due to the lower centrifugation time and the interesting juice yield [about 50% (v/v)].

Several batches of turbid juice were then produced (5 L of turbid juice per batch), resulting also the pomace (by-product).

#### Turbid Juice Crossflow Filtration Optimization

Focusing on process temperature variation, its reduction is followed by flux reduction given the viscosity-increasing of the juice, as Echavarría et al. (26) mentioned for fruit juices. This, allied with membrane fouling and concentration polarization with time, made the flux constantly reducing, as was expected. Following Pé-Leve (72) studies, the system pressure increasing (not represented), and the air backflush used may have contributed to temperature and flux stabilization with time.

M100 turbid juice used for 50°C filtration presented significantly (*p* < 0.05) more turbidity than the one used for the process starting at 60°C (Figure 3), when a similarity between them was expected. This can be explained by a possible lack of homogeneity of the turbid juice during that batch division (for tests at 60 and 50°C) and/or by natural biological differences. Therefore, a higher temperature and lower levels of solids of the 60°C condition increased the overall flow (Figure 2A) and yield, compared to the 50°C condition, probably due to lower viscosity and less retained solids (26, 35). But even considering these differences between turbid juices, the relative behavior of M100 tests were similar. Regarding M50 tests, the differences between turbid juices turbidity, were not enough to separate the flows after 75 min.

As main conclusions of this section, it can be said that: (i) there is some freedom in choosing the most convenient parameters—a lower temperature to preserve bioactive compounds and sensory attributes (27), or a higher temperature to have a better flow and a better clarified juice yield (due to lower viscosity); (ii) even if turbid juices' turbidities are significantly (*p* < 0.05) different, the process produces consistent results—clarified juice is always similar in terms of turbidity—accommodating some raw material variations (essential for industrial processes); (iii) given the high level of differences between the two turbid juices used for M100 tests, it cannot clearly be pointed the best membrane. However, small pore sizes can induce high retention of phenolics and antioxidants (73); (iv) temperature-membrane combinations need to be set considering the juice characteristics and objectives, having an impact on both resulting juices' quality and process parameters.

For the following clarified juice production, the 50 nm pore membrane was chosen, at an initial temperature of 60°C, due to previous studies stating that 100 nm pore led to faster fouling than 50 nm pore membrane tests, with “Rocha” pear juice (probably given the size of the suspended solids, which can be smaller than 100 nm, but higher than 50 nm). The 60°C initial temperature was chosen to maximize the yield.

The resulting fractions, and especially the retained fraction (rich in suspended solids), can be nutritionally interesting to develop clean label and satiating products, in a circular economy rationale. The characterization of the various fractions depends on the processing studied above and the conditions used.

From a future perspective, there is the possibility to continue the filtration optimization studies with other membranes, conditions, and equipment, to avoid losses of bioactive compounds (which can also be quantified in the various tests) and to have a better yield.

### Pear Fractions Characterization

#### Nutritional Characterization

As mentioned, all fructose values are visibly higher than other studies on pears. These differences can be due to natural biological variations. Also, the fact that the concentrated fractions are similar and richer for both sugars than the clarified juice, which is the poorest fraction, can be due to the processing procedure, given the fact that soluble compounds can be retained on suspended particles (which explain the pomace and retained fraction richness) (70) and that filtration of the turbid juice may lead to concentration polarization, retaining some glucose and fructose on the retained fraction.

As expected, given the insoluble fiber predominance in “Rocha” pear (22), pomace fraction shows significantly (*p* < 0.05) the highest content of total and insoluble dietary fiber (3.52 ± 0.100 g 100 g^−1^ and 3.43 ± 0.110 g 100 g^−1^, respectively)—puree centrifugation concentrates most of the suspended particles (which represents most of the insoluble fiber) in the pomace, as well as some soluble dietary fiber, leaving the other liquid fractions with only dietary fiber residues. Therefore, puree processing concentrates the dietary fiber in the pomace, the most suspended solids concentrated fraction, making it interesting to develop satiating and functional products.

Focusing on antioxidant activity, and regarding the puree results, some differences for the different studies of the literature can be due to puree ascorbic acid addition in the industrial line, natural biological differences among fruits and varieties, and/or differences in storage time. Focusing DPPH assay, the significant (*p* < 0.05) increase in the activity after centrifugation can be due to anthocyanins polymerization, as Tsai and Huang (74) and Guiné and Barroca (75) reported for warmed fruit juices. Also, crossflow filtration appears to not affect the antioxidant activity. About FRAP assay results, it is noticeable that pomace has significantly (*p* < 0.05) the highest activity (8.92 ± 0.498 μmol TE g^−1^), probably since it is the most concentrated fraction. The reduction of the antioxidant activity with crossflow filtration, mainly in the clarified juice (5.71 ± 0.551 μmol TE g^−1^), can be due to the degradative effect of the process—air and relatively high-temperature exposure—and/or due to bioactive compounds retained by the membrane. This reduction was also verified by Tsai and Huang (74), due to monomeric anthocyanins drop, as a result of their polymerization by heat effects. As it can be seen, there are different interpretations depending on the assay, which is consistent with the results from Kolniak-Ostek (62) in pears, reporting that the antioxidant activities were higher using FRAP assay. Although Chaves et al. (76) stated that both assays have a good correlation, several causes can be pointed, including that the DPPH method is more adequate to samples with lipophilic antioxidants or with high amounts of lipids. These authors also found out that the FRAP assay was more sensitive for plant extracts.

Moving on to total phenols results interpretation, there are several possibilities to explain the lower levels in the puree, in comparison with the other mentioned peer-reviewed studies. Once more, the possibility of the purees here being used having been stored for longer periods may well be an explanation. Also, this matrix was produced in an industrial line, which encompasses heating steps, and the pears may present some biological differences, given their campaign year, orchard, and ripening index. About the centrifugation, it is visible a reduction of the total phenols content, which can be due to the unfavorable conditions: oxygen exposure and heat (40°C), like Dutra et al. (77) mentions. The dimension of the losses is similar to Tarinoven and Eski's (67) studies for clarified pear juices. On the other hand, filtration did not affect the total phenols content, so these compounds resisted the applied conditions and passed through the membrane.

Concerning ascorbic acid results, the visible difference between pear purees from both these studies and the ones from Pedro et al. (22) is certainly due to the addition of this acid to preserve fruit purees from oxidation. The fact that the crossflow filtration had a negative impact on the resulting fractions was expected and can be explained by the unfavorable thermal conditions and the contact with the atmospheric oxygen, as it may have happened with Mai (57) who also crossflow filtrated several fruit juices, and with Mrad et al. (78) who dried pears (vitamin C thermal degradation started at 30°C).

Finally, regarding mineral composition, the pomace often presented the highest values for several minerals since it is the most concentrated fraction (in solids). The overall results, for all minerals, being higher than the values for other studies, can be explained through biological variations, as well as orchard and climate variations.

#### Physicochemical Characterization

All pear fractions produced were characterized regarding their soluble solids, pH, and turbidity parameters.

The variations on soluble solids values, for different fractions, can be explained given the processing steps that purees were subjected to: (i) some fractions of soluble solids can be adsorbed by insoluble solids, and (ii) concentration polarization effects at the crossflow filtration step can have retained soluble solids on the retained fraction. Therefore, it is normal that turbid and clarified juices and the clarified fraction are poorer in soluble solids than the by-products.

Regarding pH, it was expected that the values did not change significantly (*p* > 0.05), since processing consists mainly of suspended solids separations.

Finally, regarding turbidity, as expected, this value changed significantly (*p* < 0.05) among fractions: since they have higher amounts of suspended solids, pomace and puree fractions have the highest values of turbidity (14,476 ± 239.537l NTU and 7,010 ± 619.903 NTU, respectively). Clarified juice has, as expected, significantly (*p* < 0.05) the lowest value of turbidity (2.35 ± 0.091 NTU), since the crossflow filtration removed almost all the suspended solids.

#### Apparent Viscosity

The fact that the pomace has a significantly (*p* < 0.05) higher viscosity is due to its significantly (*p* < 0.05) lower water content, in comparison with other fractions. Therefore, its resistance to flow is higher, indicating a higher structuration level. Turbid juice and retained fraction have significantly (*p* < 0.05) lower viscosities, as expected, since they have higher (*p* < 0.05) amounts of water and fewer dry matter, in comparison with both pomace and puree, as it was observed. Regarding both turbid juice and retained fractions, the difference in the suspended solids content was not enough to produce a viscosity difference big enough to be detected by the rheometer.

### Development of a Clean Label Snack, Incorporating “Rocha” Pear Fractions, and *E. gracilis*

#### Basis-Formulation Optimization

By Figure 4 it is possible to conclude that: (i) there are no significant (*p* > 0.05) differences in using turbid juice or retained fraction in the formulations (for the incorporation percentages tested) and that (ii) the ideal retained fraction /pomace ratio to obtain the desired firmness (inside the interval delimited by the benchmarking) is 2/3 (w/w). Firmness determination was the basis for these optimizations since the viscosity tests were not effective to differentiate the small rheological differences between snack formulations.

#### Chicory Inulin Powder Addition Effect on Retained Fraction Viscosity and Snack Firmness

The variation between pasteurized and not pasteurized retained fractions with inulin is neglectable since it is only visible on the shear thinning zone of the curves (Figure 5). In the limiting infinite viscosity zone, there are no relevant (*p* > 0.05) differences between the three samples. Therefore, the effect of inulin addition and pasteurization was considered irrelevant.

Next, the incorporation of inulin in the optimized formulation (produced using the already fixed and optimized retained fraction /pomace and inulin/pomace ratios) showed that, as happened in the retained fraction tests, the inulin presence, the pasteurization, and 3 days after pasteurization does not impact significantly (*p* > 0.05) the firmness of the formulation. Therefore, inulin addition and pasteurization do not lead to any additional variation.

#### *Euglena gracilis* Incorporation

As expected, the impact of the microalgae in the formulation firmness was not significant (*p* > 0.05), comparing to the snack without microalgae. Therefore, no additional optimization was needed.

Being the final formulation fixed, it was possible to therefore produce a snack both with a firmness contained in the benchmarking range and with the possibility to have, at least, the first fiber EU nutritional claim *Source of Fiber*.

### Snack Characterization

#### Snack Physicochemical Characterization

The significant (*p* < 0.05) increase in TSS value, compared to all the pear fractions is certainly due to inulin powder addition, which contains 11 % (w/w) of sugars. Therefore, and given the pH and water results, it can be concluded that this product is vulnerable to microbial degradation (mainly fungi and lactic bacteria) (79).

Regarding color, compared with the formulation without microalgae, the lightness of the snack decreased, given the microalgae presence. The microalgae addition has a visually detectable effect, since ΔE > 5 (37). Final snack L^*^ values are higher than the benchmarking interval, but a^*^ and b^*^ are inside the respective benchmarking determined intervals.

#### Snack Nutritional Characterization

FRAP results (the ones that probably give more accurate results regarding antioxidant activity, in this situation) indicate that the processing may have a negative impact, probably given thermal polymerization and/or degradation of antioxidant compounds, like vitamin C, given the production process.

About ascorbic acid analysis, the significant (*p* < 0.05) reduction of the content of this compound (compared to pomace) may be due to, again, processing of the snack, which involves a heat treatment and contact with the atmosphere, just like happened with Petruzzi et al. (36), when produced and pasteurized fruit juices.

Regarding reducing sugars contents, the fact that both contents of glucose and fructose are significantly (*p* < 0.05) lower than the contents in the pomace and the retained fraction may be due to both pomace and retained fraction mixture and/or any process influence. Nevertheless, the difference is not very important.

It should be considered as a limitation of this study the fact that no bioactive compounds' analysis in terms of composition and bioavailability, was made. Therefore, more studies are needed to confirm what compounds are present in the snack, to subsequently understand the impact of these on human health.

### Snack Sensory Analysis

About the sensory analysis results presented in Figure 6, although very similar, since 0.12% (w/w) of *E. gracilis* was not expected to have an impact, texture evaluation for both snacks with and without this microalgae was different, something that deserves attention in future studies on the addition of *E. gracilis* to fruit products. However, these differences do not seem to be relevant for the appreciation, since several written feedbacks say that both snacks' texture is better than the commercial one.

The fact that the results from preference analysis differ from the other ones obtained (in the first, the most appreciated sample was the commercial snack, and in the other, the snack without microalgae), can be explained because when the taster is asked for an emotional response of the “like” or “dislike” type (case of the preference analysis), he may behave differently when faced with a descriptive evaluation with several attributes and with a hedonic scale of five levels (80). However, with regards to the purchase intention (Figure 7), again an advantage for the snack without microalgae is shown, as happened before (Figure 6), reinforcing the preference for this sample.

Considering these results, including the several comments written by the panelists, the formulation without microalgae was the most accepted, being the one that individuals did have the highest intention to buy, overcoming the commercial one.

Therefore, the sensory improving point for the aimed snack is the optimization of *E. gracilis* content (which would not be beneficial to decrease since it is already low) or the flavor and aroma smoothing. The final snack (with microalgae) flavor and aroma disadvantages can eventually be overcome by (i) adding spices (like cinnamon, for example); (ii) mixing with cereals (like is already done with the used commercial sample) and/or (iii) mixing it with yogurt. Naturally, some of these options would change the nutritional value and food safety parameters, but it could be a future interesting study, along with the sensory optimization.

### Snack Nutritional Claims

Consulting the approximate composition of the snack (Table 6) and the legislation, the EU nutritional claims that may be interesting to include on the label, to increase the attractiveness of the product, are: *Fat Free*; *High Fiber*; *Source of Iron*; *Source of Copper*; *Energy-Reduced*; *It contains dried biomass of Euglena gracilis algae*.

Also, other non-regulated claims that may be interesting to show on marketing communications are *Plant and microalgae-based product*, and *Clean Label*, given the proven increasing demand for clean labels, microalgae consumption, and plant-based products.

Finally, it is important to mention that the developed snack has only 63% of the energetic value from the commercial sample tested in the sensory analysis (*Compal à colher de pera e ananás*), and more circa 2% (w/w) of dietary fiber, which are better characteristics comparing with the mentioned product.

## Conclusion

With this study we accomplished the (i) valorization of pear by-products, in a circular economy rationale, developing a clean label snack with superior dietary fiber content, incorporating *E. gracilis*; (ii) turbid and clarified pear juices laboratory production optimization; (iii) physicochemical, rheological, and nutritional characterization of both the developed pear snack and the several pear fractions.

The optimization processes resulted in preferred conditions − 270 s/2,600 × *g*/40°C to centrifuge the puree, and 50 nm pore membrane/60°C initial temperature to filtrate the turbid juice. The fractions analysis mainly highlights the particularly high content in ascorbic acid (resisting the centrifugation step) and dietary fiber of the pomace and the puree; the impoverishment in the dietary fiber content of the juices; the total phenols resilience to the filtration; and the antioxidant activity resistance to the centrifugation.

Regarding the developed functional liquid snack, it has around 4.51% (w/w) of dietary fiber, the total phenols resisted the processing, but the ascorbic acid was below the detection limit. The developed snack (with microalgae) was relatively well-accepted by the untrained panel of 51 individuals, mainly in texture and general appearance aspects. Nevertheless, it has the potential for sensory improvement, mainly in terms of the flavor and aroma given by the microalgae, since the formulation without microalgae won in most parameters. Until now, this is an innovative clean label product, which valorizes a Portuguese pear variety and its by-products in a circular economy rationale, incorporating an approved novel food (*E. gracilis*), being plant-based and rich in dietary fiber, and likely to have an immunomodulator, satiating and prebiotic effect. Also, this product may be especially interesting for children and elderly people, because of the dietary fiber content with a relevant impact on the gut microbiome, which is important to children's healthy development and healthy aging for the elderly (fighting aging mechanisms and osteoporosis, for example). Additionally, this product allows EU nutritional claims, which can be advantageous both in economical and health aspects.

This work not only proposed a use for juices' industry by-products but also contributed to characterizing several pear fractions more widely. It can be continued by further optimizing the turbid juice filtrations (e.g., with other membrane pores and materials) to have a faster and profitable process, study the bioactive compounds' composition of the snack and the fractions, study the snack' shelf-life, test the satiating, immunomodulatory and prebiotic effects of this product, and finally, optimize its sensory characteristics.

## Data Availability Statement

The original contributions presented in the study are included in the article/supplementary material, further inquiries can be directed to the corresponding author/s.

## Ethics Statement

Ethical review and approval was not required for the study on human participants in accordance with the local legislation and institutional requirements. The patients/participants provided their written informed consent to participate in this study.

## Author Contributions

IS and AR contributed to problem formulation, design of the study, the developed snack concept, and the discussion of the results. XL-V contributed to problem formulation, study, conception, developed product design, statistical analysis, and writing the first draft of the manuscript. CP contributed mainly to the centrifugation optimization study, in several laboratory processes and data analysis. MA contributed by giving SUMOL+COMPAL technical and business information and facilitating purees supplying, also being initially involved in the developed snack concept. All authors contributed to manuscript revisions, read, and approved the submitted version.

## Funding

This work was supported by Agência Nacional de Inovação (Portugal) through the project *cLabel*+ (PO CI/ANI/46080/2019). Financial support was also acknowledged to FCT through Research Unit LEAF (UIDB/04129/2020) funding. It is also acknowledged to Banco Santander Universidades/Instituto Superior de Agronomia the masters financial support, through Prémio de Incentivo ao Mestrado – Edição 2019.

## Conflict of Interest

MA was employed by the company SUMOL+COMPAL. The remaining authors declare that the research was conducted in the absence of any commercial or financial relationships that could be construed as a potential conflict of interest.

## Publisher's Note

All claims expressed in this article are solely those of the authors and do not necessarily represent those of their affiliated organizations, or those of the publisher, the editors and the reviewers. Any product that may be evaluated in this article, or claim that may be made by its manufacturer, is not guaranteed or endorsed by the publisher.

## Abbreviations

SS: Suspended Solids
M50: Ceramic membrane with a pore diameter of 50 nm
M100: Ceramic membrane with a pore diameter of 100 nm
EU: European Union.
